# Low-cost oscillating sieving shaker machine for granulometry analysis in food science

**DOI:** 10.1016/j.ohx.2025.e00736

**Published:** 2025-12-21

**Authors:** Juan Carlos Núñez Dorantes, Mario Luna Flores, José Roberto Grande Ramírez, Verónica Flores Sánchez, Jonathan Josue Cid Galiot, José Ernesto Domínguez Herrera

**Affiliations:** Postgraduate Department at the Universidad Tecnológica del Centro de Veracruz, Av. University, 350, 94910 Cuitláhuac, Veracruz, Mexico

**Keywords:** Low-cost hardware, Oscillating sieve shaker, Particle size distribution, Food powder analysis, 3D printing, Finite Element Analysis (FEA)

## Abstract

This article presents the design, fabrication, and validation of a low-cost oscillating sieving shaker machine developed for laboratory-scale granulometric analysis of powdered and solid food materials. The system integrates 3D-printed PLA components, an aluminum modular frame, and a dual gear-motor mechanism that generates controlled oscillatory motion for particle size classification. Designed under open-source and affordability principles, the device was constructed using locally available materials and standard FDM-printing parameters. Finite Element Analysis (FEA) of critical components printed in PLA, PETG, and ABS confirmed safe elastic behavior under a representative 5 kg load, with stresses below 21 MPa and displacements under 1.7mm. A thermal-impact study established a linear correlation between load, motor current, and temperature (R^2^ ≈ 0.99), with a maximum temperature of 28.8 °C—well below the glass-transition limits of PLA and PETG—ensuring thermally stable operation. Performance tests performed according to ASTM C136/C136M-19 on soy lecithin, potato starch, and ascorbic acid confirmed accurate and reproducible particle-size distributions consistent with literature values. The total fabrication cost of USD 143.78 represents a significant reduction compared to commercial shakers (USD 2,800–3,700). This work validates an open-source, cost-effective, and reproducible device suitable for educational and research laboratories requiring reliable granulometric control in food powder analysis.

## Graphical abstract

**Figure:**
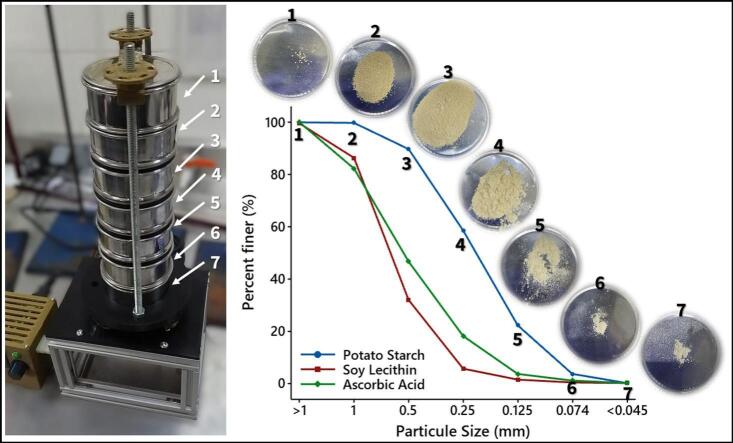

**Specifications table**

**Table:** 

Hardware name	*Oscillating sieving shaker machine*
Subject area	• Engineering and materials science • Food Sciences
Hardware type	• Mechanical engineering and materials science
Closest commercial analog	*Sieve Shakers*
Open source license	Creative Commons Attribution 4.0 International (CC BY 4.0)
Cost of hardware	*143.78 dollars*
Source file repository	*https://doi.org/10.17605/OSF.IO/CZ4VW*

## 1 Hardware in context

Particle size distribution has a fundamental role in food science, as it influences processing behavior, solubility, water absorption, texture, emulsions stability, and even nutritional and sensory properties. For example, finer particles can improve bioavailability and modify flavor release and mouthfeel, while coarse particles affect flowability and texture. Due to these multifaceted roles, accurate and reliable granulometric analysis is crucial for ensuring quality control and driving innovation in food processing. Among the different techniques available, sieve analysis remains one of the most widely applied methods due to its simplicity, reproducibility, and direct relevance to industrial processes [1,2].

Commercial oscillating sieve shakers provide accurate particle size classification, but their high cost (typically USD 2800–3700, excluding sieves) restricts their use in educational institutions, small laboratories, and developing regions. To address these limitations, various low-cost designs have been reported. For instance, in soil science, vertical oscillation systems have been developed to determine aggregate stability with reliable results [3]. Similarly, rotary vibration-based sieving has been proposed for soil classification [4], low-cost mechanical sieve shakers have been fabricated for ceramics [5], and automatic versions have been introduced for educational purposes, including tools for visually impaired students [6]. While these efforts demonstrate the feasibility of low-cost solutions, they often require advanced fabrication skills, omit detailed build instructions, or lack validation in food-related applications.

In food science specifically, the necessity for cost-effective sieving equipment is even more pronounced. Particle size directly impacts wheat flour quality, influences solubility and morphology in powdered ingredients [1], and determines stability in beverages and sauces. Yet, despite the central role of granulometric control [2], there is still no openly available, reproducible, and low-cost oscillating sieve shaker specifically designed and validated for food powders.

To address this gap, a laboratory-scale oscillating sieving shaker machine is introduced for granulometric analysis of powdered and solid food materials, integrating 3D-printed PLA components, an aluminum modular frame, and a dual-gear motor system to produce controlled oscillatory motion. The design is fully open-source, affordable, and reproducible with locally available materials.

## 2 Hardware description

**Fig. 1:**
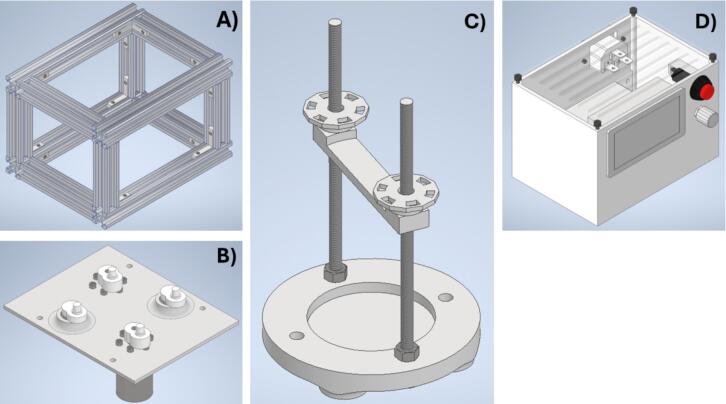
Main structure (A), drive shaft (B), sieve support (C), and electronic assembly container (D) of oscillating sieving shaker machine.

The oscillating sieving shaker machine was designed to be fabricated in four distinct phases. The first phase involves the construction of the main structure (Fig. 1A), the second focuses on the assembly of the drive shaft (Fig. 1B), the third consists of integrating the sieve support system (Fig. 1C), and the last phase focuses on the assembly of the electronic components (Fig. 1D).

The main structure was assembled using aluminum profiles joined by L-shaped aluminum extrusion connector brackets secured with screws and a wrench. This modular design allows for easy modification; for instance, if an increase in the diameter of the drive shaft or sieves is required, Adjust in the length of the aluminum profiles should be done.

The drive shaft transmits the driving force to actuate two eccentric cams, which generate the oscillating motion of the shaker through the use of two gear motors. An additional pair of eccentric cams, supported by ball bearings, guides the linear movement of the sieve support with precision. All components are mounted on the drive shaft base, which ensures structural integrity and proper alignment during operation. The implementation of eccentric cam mechanisms in this configuration is critical for transforming rotational input into controlled oscillatory motion. Similar mechanisms have been validated in laboratory-scale equipment, such as the cam profile measuring machine [7], which demonstrated the effectiveness of various cam profiles in producing reliable and repeatable follower displacements in mechanical systems and in the biomedical field, particularly in automating repetitive procedures associated with Western blot processing[8]. These precedents highlight the versatility and functional reliability of eccentric cams in generating precise motion in scientific and engineering applications.

The sieve support secures the sieves between the bottom sieve holder and the upper sieve clamp. This assembly is fitted onto the eccentric cams of the drive shaft, enabling the transmission of oscillatory motion to agitate the sieves effectively. The sieve support system is specifically designed to accommodate 3-inch sieves. To enable the use of larger sieves, such as those measuring 8 in. in diameter, structural modifications would be required to the drive shaft base, sieve bottom support, and upper sieve support components.

Additionally, the sieve support system can accommodate both stainless-steel and polymeric (nylon) sieves that comply with ASTM E11 standards. Stainless-steel sieves are preferred when durability and dimensional stability are required, whereas nylon sieves are advantageous for hygroscopic or electrostatically sensitive powders. The modular clamping mechanism allows quick substitution between different sieve materials without the need to redesign or reprint structural components, thus enhancing the adaptability of the device for diverse analytical applications.

The electronic components were assembled within an enclosure to ensure the protection and secure integration of all elements. Externally, the enclosure features a power switch for activating and deactivating the device, a potentiometer for adjusting the rotational speed (RPM), a display screen for monitoring the RPM, a voltage input socket, and a Molex female connector for interfacing with the motor. This configuration enables effective control of various 12 V gear motors. Accordingly, it is suitable for diverse applications, such as regulating the RPM of the collector in the Solution Blow Spinning technique [9], controlling the Scientific Bottle Roller [10], operating peristaltic pumps [11], and other scientific instrumentation.

## 3 Design files summary

The components were initially designed in Autodesk Inventor 2024 (the free student and educator version). Once completed, the designs were exported in Surface Tessellation Language (*.stl) format to represent the three-dimensional surface geometry. These STL files were subsequently processed in CURA to generate the G-code required for 3D printing.

The manufacturing process was conducted using a Kingroon KP3S 3D printer, which operates with Fused Deposition Modeling (FDM) technology. Polylactic acid (PLA) filament was used as the printing material, extruded through a 0.4 mm-diameter nozzle at 200 °C for the extruder and 60 °C for the bed. Printing parameters included a layer height of 0.2 mm, a print speed of 80 mm/s, and a 50 % infill density utilizing a lines infill pattern. This pattern was selected based on reports indicating its association with higher Young’s moduli and reduced variation in Poisson’s ratio in FDM-fabricated PLA components [12]. PLA was chosen due to its broad availability, ease of processing, and favorable mechanical properties under optimized printing conditions, which support its applicability in functional prototyping[13].

The Description of Printable Components section provides a comprehensive overview of the function and role of each 3D-printed component required for the assembly of the oscillating sieving shaker machine. The corresponding STL and IPT file locations are available in the source file repository and are distributed under the Creative Commons Attribution 4.0 International (CC BY 4.0) open-source license, as shown in Table 1.

**Table 1:** Design files summary.

**Design filename**	**File type**	**Open-source license**	**Location of the file**
Eccentric cam	IPT & STL	CC BY 4.0	https://osf.io/cz4vw/
Drive shaft base	IPT & STL	CC BY 4.0	https://osf.io/cz4vw/
Sieve bottom support	IPT & STL	CC BY 4.0	https://osf.io/cz4vw/
Upper sieve support	IPT & STL	CC BY 4.0	https://osf.io/cz4vw/
Handwheel	IPT & STL	CC BY 4.0	https://osf.io/cz4vw/
Container for the control system	IPT & STL	CC BY 4.0	https://osf.io/cz4vw/
Container lid	IPT & STL	CC BY 4.0	https://osf.io/cz4vw/
Eccentric cam with housing for drive shaft	IPT & STL	CC BY 4.0	https://osf.io/cz4vw/

**Description of printable components.**

1. Eccentric cam. Converts the rotational motion of the drive shaft into oscillatory motion. It is crucial for generating the lateral vibrations necessary to agitate the sieves during granulometric analysis effectively.
2. Drive shaft base.- Provides the structural support and alignment for the rotating drive shaft and attached components. It ensures mechanical stability and transmits the mechanical loads induced by oscillatory motion.
3. Sieve bottom support.- Serves as the base platform for holding the stack of sieves in position during testing. It is directly connected to the oscillatory mechanism and must ensure stable and uniform agitation.
4. Upper sieve support.- Functions as the top clamp for securing the sieves within the assembly. It presses the sieve stack against the bottom support to maintain alignment and prevent displacement during vibration.
5. Handwheel.- Allows manual tightening or adjustment of the upper sieve support. It ensures that the sieves are firmly secured for accurate and consistent testing.
6. Container for the control system.- Houses all electronic components necessary for operating the machine, including the power switch, potentiometer, display screen, voltage input, and motor connection ports. It provides protection and safe integration of the control system.
7. Container lid.- Covers and protects the internal electronics housed within the control system enclosure. It ensures safety and maintains the integrity of the electronic assembly during operation.
8. Eccentric cam with housing for drive shaft.- This component integrates the functionality of an eccentric cam with a housing structure designed to facilitate secure mounting onto the gear motor. It transforms the rotational force generated by the gear motor into controlled oscillatory motion, which is essential for the agitation of the sieve assembly.

## 4 Bill of materials summary

### 4.1 Printable materials requirements

A total of eight 3D-printable components were required for the construction of the oscillating sieving shaker machine. The weight of each component, fabricated using PLA filament, along with the corresponding printing time, is presented in Table 2. A total of 436 g of PLA filament was used for fabrication; however, accounting for material loss during pre-print adjustments and calibration, an estimated 500 g of PLA was considered. The total printing time for all components amounted to 2,190 min, equivalent to 36 h and 30 min.

**Table 2:** Design Files Summary and PLA requirements.

**No**	**Design file name**	**PLA weight (g)**	**Time to print (min)**
1	*Eccentric cam*	*13*	78
2	Drive shaft base	*100*	471
3	Sieve bottom support	*130*	560
4	Upper sieve support	*17*	99
5	Handwheel	*11*	83
6	Container for the control system	*127*	667
7	Container lid	*24*	147
8	Eccentric cam with housing for drive shaft	*13*	85
		435	2190

The bill of materials required for the assembly of the oscillating sieving shaker machine is presented in Table 3. It includes the name of each component, quantity, unit of measurement, cost per unit, total cost per component, and the overall cost. Although some components were purchased in packages, the unit cost was calculated by dividing the total price of the package by the number of units it contained.

**Table 3:** Bill of materials.

**No**	**Component**	**Quantity**	**Unit**	**Cost per unit**	**Total cost (USD)**
1	PLA Filament	0.5	Kg	$ 13.99	$ 7.00
2	M8 Hex Nut	6	Item	$ 0.25	$ 1.49
3	M5 T Nuts	4	Item	$ 0.35	$ 1.40
4	L-Shape Aluminum Connectors	16	Item	$ 0.58	$ 9.33
5	Ball Bearing	8	Item	$ 0.99	$ 7.92
6	5/16 Threaded Rod	2	Item	$ 4.99	$ 9.98
7	Panel Mount Power Plug	1	Item	$ 0.99	$ 0.99
8	Gear Motor High Torque Electric	2	Item	$ 14.99	$ 29.98
9	Power Supply Adapter Universal	1	Item	$ 12.99	$ 12.99
10	Aluminum linear rail	1.5	Meter	$ 19.99	$ 29.99
11	DC Motor Speed Controller	1	Item	$ 10.99	$ 10.99
12	AC Power Cord Cable	1	Item	$ 6.09	$ 6.09
13	Molex cable	1	Item	$ 9.99	$ 9.99
14	20 AWG PVC Electrical Wire	2.5	Meter	$ 1.34	$ 3.35
15	M5 Cap Screws	4	Item	$ 0.09	$ 0.36
16	M3 Cap Screws	16	Item	$ 0.11	$ 1.76
17	M4 Cap Screws	2	Item	$ 0.10	$ 0.20
				**TOTAL**	**$ 143.78**

Material costs were obtained from the Amazon online store. To ensure accurate product identification, the Amazon Standard Identification Number (ASIN) and specification for each item are included in the table called “Bill of materials” available in the source file repository.

The total cost of the components required for assembling the oscillating sieving shaker machine amounted to $143.78 USD. This figure represents the unit fabrication cost. In comparison, commercial laboratory-grade sieve shakers typically range from $2,800 to $3,700 USD, excluding the cost of sieves. Therefore, the designation of the developed device as a low-cost alternative is well justified.

## 5 Build instructions

The building instructions were organized into phases. The first phase outlines the assembly of the main structure; the second focuses on the assembly of the drive shaft; the third details the construction of the sieve support; and the fourth phase addresses the electronic assembly, and finally, the full assembly is shown. Although each phase is described within the article, visual assembly procedures are also available in the source file repository for further reference.

### 5.1 Main structure assembly

1. Cut the aluminum linear rail into four sections of 180 mm, four sections of 104 mm, and four sections of 80 mm.
2. Arrange two 180 mm rails and two 104 mm rails on a flat surface to form a rectangular base. Insert L-shaped aluminum connectors at each corner to join the rails at 90° angles.
3. Align the rails and tighten the connectors using grub screws, ensuring all joints are secure.
4. Repeat Steps 1 to 3 to assemble a second identical rectangular base.

After securing all components, verify that the frame forms a perfect rectangle and that all corners are properly aligned. Similarly, ensure that all connections are firmly tightened to guarantee structural stability for the subsequent vertical assembly. A demonstrative video is available in the source file repository under the title Main Structure Assembly 1.

5. Insert L-shape aluminum connectors at each corner and attach the four 80 mm rails vertically to each corner of the first base using the available connector slots, and repeat the frame assembly using the second rectangular base.
6. Finally, align the rails and tighten the connectors using grub screws, ensuring all joints are secure

Inspect the completed cubic frame for proper alignment and squareness. Re-tighten all grub screws to finalize the assembly of the main structure. A demonstrative video is available in the source file repository under the title Main Structure Assembly 2. The assembled main structure is illustrated in Fig. 1A.

### 5.2 Drive shaft assembly

**Fig. 2:**
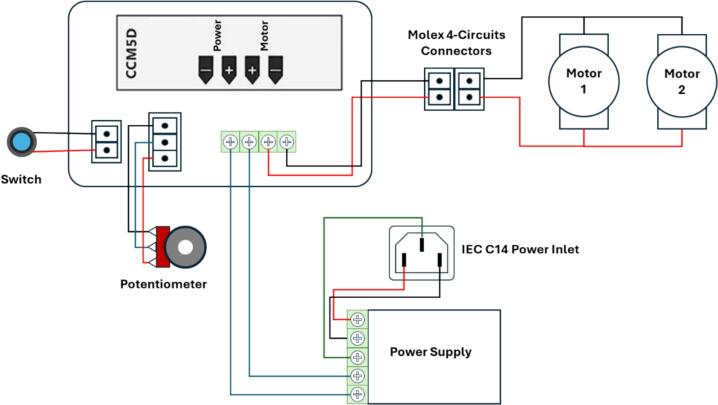
Wiring diagram of electronic components.

1. Align each high-torque gear motor with the designated mounting position on the drive shaft base, ensuring that the motor shaft fits precisely into the corresponding hole.
2. Secure the gear motors to the structure using M3 cap screws, maintaining proper alignment and ensuring a firm attachment.
3. Insert the bearings into their respective slots or holders on the base. Confirm that they are correctly aligned and securely press-fitted or fixed in place.
4. Insert the eccentric cam shafts into the mounted bearings.
5. Connect each eccentric cam (with housing for drive shaft) to the shaft of the gear motor by inserting the motor shaft into the corresponding hole on the eccentric cam. Repeat this process for both motor shafts and eccentric cams.
6. Ensure that the eccentric cams are aligned in the same orientation.
7. Connect the gear motors to the male Molex connector in accordance with the wiring diagram shown in Fig. 2.

The assembly procedure is demonstrated in the video titled *“003 Drive Shaft Assembly.wmv”*, available in the source file repository. Nevertheless, the final configuration of the assembled drive shaft is illustrated in Fig. 1B.

### 5.3 Sieve support assembly

1. Thread an M8 hex nut onto the lower section of the rod to properly adjust and secure the position of the sieve bottom support.
2. Insert the 5/16″ threaded rod vertically through the center of the sieve bottom support, allowing it to pass upward through the entire structure.
3. Thread an additional M8 hex nut onto the upper end of the rod until it reaches and firmly secures the upper sieve support in place.
4. Install the ball bearing into its designated holder within the bottom support to facilitate smooth rotation of the threaded rod. Ensure that the bearing is properly seated and securely fixed in place.
5. Guide the threaded rod through the upper sieve support.
6. Thread an M8 hex nut onto the upper part of the rod until it secures the upper sieve support.
7. Affix the handwheel onto the upper M8 hex nut and apply adhesive to ensure a secure and permanent attachment.

The final configuration of the assembled sieve support system is illustrated in Fig. 1C, while the step-by-step assembly procedure is demonstrated in the video titled “004 Sieve Support System Assembly”, available in the source file repository.

### 5.4 Electronic assembly

1. Insert the 12 V power supply adapter into the designated space within the control system container.
2. Mount the DC motor speed controller onto the front panel of the container, ensuring that its user interface remains accessible from the exterior.
3. Install the potentiometer through the assigned opening and secure it in place using a hex nut. Finally, attach the knob to the potentiometer shaft.
4. Mount the power switch button in its corresponding location and fasten it using the appropriate hex nut.
5. Secure the panel mount power plug into the designated cutout of the container using M4 cap screws, ensuring a firm attachment.
6. Affix the Molex female connector to the side wall of the container using industrial-grade adhesive to prevent displacement during operation.
7. Use 20 AWG PVC electrical wire to connect all input and output terminals according to the wiring diagram shown in Fig. 2. Ensure correct polarity and terminal alignment.
8. Conduct a visual inspection to confirm that all electrical connections are secure, properly insulated, and free from exposed wiring.
9. Close the control system container with the container lid and fasten it using M3 screws.
10. Finally, connect the AC power cord to a reliable power source and verify that the DC motor speed controller functions as intended.

Although the electronic assembly process is demonstrated in the video titled “005 Electronic Assembly”, available in the source file repository, the final configuration is illustrated in Fig. 1D.

### 5.5 Final Assembly of the Oscillating Sieving Shaker Machine

1. Position the main structure securely on a stable base.
2. Place the M5 T-nuts into their designated slots on the structure to accommodate the drive shaft assembly.
3. Install the drive shaft in its proper position and secure it using M5 screws.
4. Mount the sieve support system by carefully inserting the eccentric shafts into the central holes of the bearings, ensuring proper alignment and fit (Fig. 3).

**Fig. 3:**
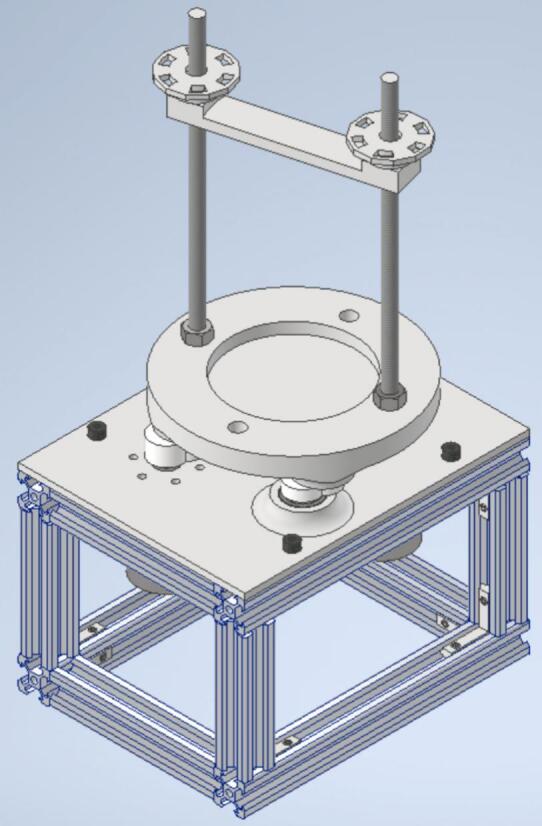
Final Assembly of the Oscillating Sieving Shaker Machine.
5. Connect the gear motor to the electronic components using the Molex connectors, as illustrated in Fig. 2.

The assembly procedure of the oscillating sieving shaker machine is documented in the source file repository through the video titled “006 Final Assembly”. Similarly, the complete assembly of the machine is illustrated in Fig. 3.

## 6 Operation instructions

1. Place the oscillating sieving shaker machine securely on a stable and level surface.
2. Measure sieves individually.
3. Arrange the sieves in a stacked (tower) configuration, ordering them according to the desired mesh opening size.
4. Position the sieves onto the sieve bottom support, ensuring proper alignment.
5. Place the upper sieve support on top of the stacked sieves and use the handwheel to apply downward pressure and secure them in place.
6. Connect the power cord cable to the panel mount power plug, and then connect the system to the electrical supply.
7. Set the potentiometer to its lowest setting prior to activation.
8. Use the power switch button to turn on the machine.
9. Adjust the potentiometer to regulate the desired rotational speed (RPM), considering that 100 % power corresponds to approximately 150 RPM.
10. Allow the machine to operate for the required duration as defined by the experimental design.
11. Once the process is complete, switch off the device using the power switch button.
12. Carefully remove the sieves and measure the weight of the material retained in each sieve.

The operating parameters of the oscillating sieving shaker—frequency of approximately 150 RPM, sieving duration of 30 min (it represents a maximum validation time, not a mass-dependent requirement), and maximum load of 2 kg—were selected in accordance with the procedures described in ASTM C136/C136M-19 for dry sieve analysis. These conditions ensure representative particle separation without overheating or clogging and can be adjusted based on the sample type, as detailed in the performance validation section.

## 7 Validation and characterization

### 7.1 Finite element analysis of printable components

To assess the structural integrity of the 3D-printed components of the low-cost oscillating sieving shaker machine under operational conditions, a finite element analysis (FEA) was conducted. This method was selected based on its proven ability to yield results closely aligned with experimental data, as reported in previous [14]studies [14,15]. The analysis focused on the eccentric cams, shaft base, and sieve bottom support—components subjected to mechanical loads resulting from the weight of the sieves and the material being analyzed.

### 7.2 Force and pressure estimation

The applied forces and pressures were determined based on the average weight of the sieves and sample material used during testing. The total force (in Newtons) applied to each component was calculated using Eq. (1):

(1) $$Force\left(N\right)=\left(mass\right)\left(g\right)$$

Where:

• *F* is the force in Newtons (N),
• *m* is the total mass (kg) comprising the sieves and average sample material,
• *g* is the gravitational constant (9.81 m/s^2^).

Subsequently, the pressure applied to specific contact zones of the components was calculated using Eq. (2):

(2) $$\mathrm{Pressure}=\frac{\mathrm{Force}}{\mathrm{area}}$$

Where:

• *P* is the pressure in Pascals (Pa),
• *F* is the force (N),
• *A* is the contact area (m^2^) over which the force is distributed.

These calculated values were used as input parameters in the FEA simulations to verify whether the selected components could sustain the operational loads without mechanical failure.

### 7.3 Mass assumptions for finite element analysis

For an ASTM E11 test sieve with a diameter of 3 and 8 in., featuring a stainless-steel mesh and a full-height stainless steel frame, the weight typically ranges between 300 and 500 g. Sieves with different frame heights will exhibit variations in weight accordingly.

Although the oscillating sieving shaker machine developed in this study was originally designed for 3-inch sieves, the parameters used in the finite element analysis (FEA) considered the maximum load associated with 8-inch sieves. This approach ensures that the machine can be subsequently modified to accommodate 8-inch sieves, guaranteeing proper functionality and structural integrity under increased operational loads.

For the finite element analysis (FEA), a total load of 5 kg was considered to simulate the operational conditions of the oscillating sieving shaker machine. This value was established based on the following components: five sieves, each weighing approximately 0.5 kg (totaling 2.5 kg), a stainless-steel container and sieve lid contributing an additional 0.5 kg, and 1.0 kg of sample material representing the average quantity processed during each test.

It is important to note that standard testing norms do not explicitly define the amount of material to be loaded, as the volume capacity of the top sieve depends on the density of the material being analyzed. Therefore, 1.0 kg of sample material was adopted as a representative value. To ensure the reliability and safety of the design, a final load assumption of 5.0 kg was used for the FEA simulations.

### 7.4 Structural validation of 3D-printed components

To assess the mechanical viability of the low-cost oscillating sieving shaker machine, a finite element analysis (FEA) was conducted on key 3D-printed components fabricated from commercial filaments as Polylactic Acid (PLA), Polyethylene Terephthalate Glycol-modified (PETG), and Acrylonitrile Butadiene Styrene (ABS). The components analyzed included the eccentric cam, the eccentric cam with housing for the drive shaft, the drive shaft base, and the sieve bottom support. The mechanical properties of different filaments used in the simulations are summarized in Table 4.

**Table 4:** Properties of printable PLA, PETG, and ABS filaments.

**Property**	**PLA**	**PETG**	**ABS**
Mass density (g/cm^3^)	1.25	1.541	1.06
Yield strength (MPa)	35	54	20
Ultimate tensile strength (MPa)	50	55.1	29.6
Young’s modulus (GPa)	3	2.758	2.224
Poisson’s ratio (su)	0.4	0.42	0.38
Shear modulus (GPa)	1.2	1.24	0.85

A comparative finite-element analysis (FEA) was performed to evaluate the mechanical integrity of the principal 3D-printed components—sieve bottom support, eccentric cam, eccentric cam with housing for the drive shaft, and drive-shaft base—when fabricated in PLA, PETG, and ABS. Each virtual prototype was subjected to a representative total load of 5 kg, corresponding to the combined mass of five sieves, the container, and the test material, with a 25 % safety margin included. Maximum Von Mises stress (σVM, max), displacement, and Safety factor (FS) values for each printable component and material are presented in Table 5.

**Table 5:** FEA results for printable components.

**Component**	**Material**	**σVM,max (MPa)**	**Displacement (mm)**	**FS**
**Sieve Bottom Support**	ABS	7.6	0.28	2.63
	PETG	7.16	0.221	**7.59**
	PLA	7.38	0.206	4.74
**Eccentric Cam**	ABS	8.03	0.022	2.49
	PETG	7.93	0.022	**6.86**
	PLA	7.98	0.02	4.39
**Eccentric Cam + Housing**	ABS	4.86	0.021	4.12
	PETG	4.78	0.017	**11.38**
	PLA	4.82	0.016	7.27
**Drive Shaft**	ABS	21.4	0.172	0.93
	PETG	20.5	1.692	**2.66**
	PLA	20.9	1.587	1.67

The calculated maximum Von Mises stress, total displacement, and safety factor values are summarized in Table 4. All polymers remained below their respective yield strengths—approximately 53 MPa for PLA, 50 MPa for PETG, and 40 MPa for ABS—confirming purely elastic behavior under service conditions. PETG displayed the highest safety factors (6.86–11.38), followed by PLA (4.39–7.27) and ABS (2.49–4.12). These differences are consistent with experimental tensile data reported by Menargues et al. [16], who observed Young’s moduli of 3.5 GPa, 2.0 GPa, and 2.1 GPa for PLA, PETG, and ABS, respectively, confirming that PLA is stiffer, whereas PETG is more ductile and capable of dissipating localized stresses through plastic deformation.

The sieve-bottom support exhibited maximum stresses of less than 8 MPa for all materials. PLA and PETG achieved the lowest displacements (0.206 mm and 0.221 mm), indicating sufficient rigidity to maintain the horizontal alignment of the sieve stack. ABS, though serviceable, presented the lowest safety factor (2.63), reflecting its higher susceptibility to creep and fatigue under cyclic loading. For the eccentric cam assemblies, responsible for transforming rotation into oscillatory motion, PETG provided the largest safety margin (FS ≈ 6.9–11.4), while PLA maintained the smallest deflection (< 0.022 mm), ensuring repeatable oscillation amplitude with minimal vibration loss.

In the drive-shaft base, the region of greatest bending and torsional stress, all polymers registered σ_VM ≈ 20–21 MPa. PETG achieved the highest safety factor (2.66) due to its superior elongation at break, whereas PLA exhibited the lowest displacement (1.587 mm), which is desirable for maintaining dimensional accuracy and alignment. These numerical tendencies coincide with the experimental evidence that PETG’s higher ductility allows it to absorb strain energy more efficiently, whereas PLA’s crystallinity and higher modulus result in reduced deformation under identical loading[16].

Despite PETG’s larger theoretical safety factor, PLA was selected as the standard fabrication material because it provides the most favorable compromise between stiffness, dimensional precision, and printability. As documented by Menargues et al. (2025) and corroborated by Hsueh et al. (2021), PLA maintains optimal interlayer bonding and mechanical strength when printed near 210–230 °C, whereas ABS suffers degradation above 220 °C and PETG often exhibits thermal relaxation or stringing above 230 °C[16,17]. Furthermore, PLA can be processed at lower extrusion temperatures (∼200 °C) without requiring an enclosed printer, minimizing warping and ensuring consistent geometric fidelity [18]. Its biobased origin, low emission of volatile compounds, and food-contact safety make it particularly suitable for academic and food-science laboratories.

**Fig. 4:**
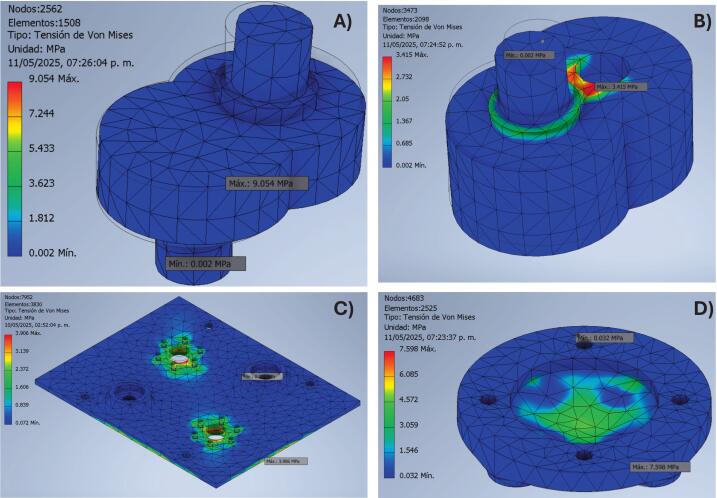
FEA Analysis of Eccentric cams without (A) and with housing for drive shaft (B), Drive shaft base (C), and Sieve bottom support (D).

The comparative FEA confirms that all three materials can safely withstand operational loads without structural failure. PETG exhibits the highest resilience and damping capacity, ABS the lowest stiffness and dimensional stability, and PLA the best balance between rigidity, print reliability, and environmental compatibility. The predicted stresses (≤ 21 MPa) and minimal displacements (< 1.7 mm) explain the stable oscillatory performance observed experimentally during granulometric testing. The strong agreement between simulation and experimental results demonstrates that the selected PLA configuration ensures mechanical reliability, cost efficiency, and reproducibility using open-source fused-filament fabrication platforms. The distribution of stress and deformation across the evaluated components is illustrated in Fig. 4.

Although PLA is considered biocompatible and food-safe, its hydrolytic sensitivity may lead to gradual reduction in tensile strength and stiffness when exposed to humid environments. To extend operational life, protective coatings or periodic replacement of high-stress components (such as cams and supports) are recommended, particularly in laboratories where humidity levels are high.

### 7.5 Thermal impact

To verify whether heat generation could influence the structural integrity of the 3D-printed components, a thermal impact evaluation was performed by correlating the applied load (g) with the electrical current (A) and surface temperature (°C) of the gear motors—the primary sources of heat during operation. The oscillating sieving shaker was tested at full speed (≈150 RPM) while progressively increasing the total load from 1113 g to 2000 g in 250 g increments. Current and temperature were measured after 20 min of continuous operation under each condition.

**Fig. 5:**
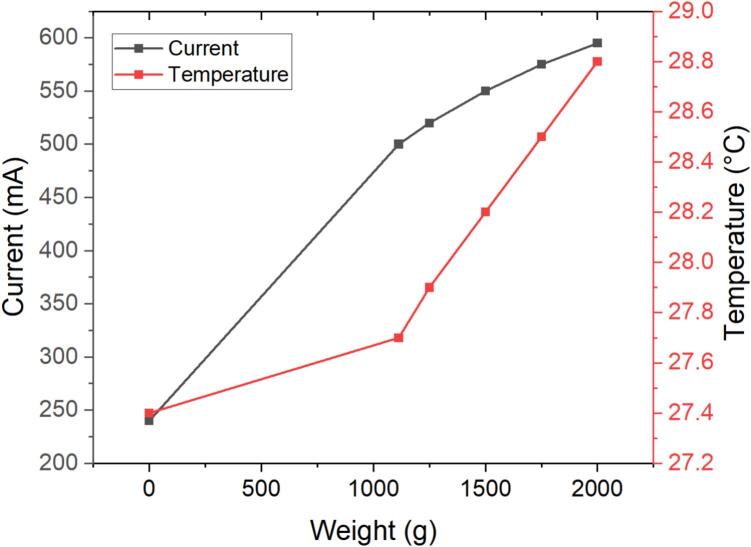
Relationship between load, electrical current, and motor temperature.

As illustrated in Fig. 5, both current and temperature increased linearly with weight. The current rose from 0.50 A at 1113 g to 0.60 A at 2000 g, indicating a proportional rise in mechanical demand on motors. Likewise, the motor surface temperature increased gradually from 27.7 °C to 28.8 °C with a high linear correlation (R^2^ ≈ 0.99). The temperature change of only 1.1 °C across the entire load range demonstrates excellent thermal stability. The observed heating originates from resistive losses in the motors rather than friction or deformation of the printed parts.

The maximum temperature remained far below the glass-transition limits of the polymers used in fabrication—approximately 60 °C for PLA and 70 °C for PETG[16]—confirming that the oscillating sieving shaker operates under thermally safe conditions. Therefore, no heat-induced warping or softening of the 3D-printed components is expected during normal or extended operation.

### 7.6 Sieving performance evaluation

Although the structural design considered a representative load of 1 kg for safety validation, this value refers exclusively to the mechanical requirements used in the finite element simulations. It does not correspond to the amount of material used during experimental testing. To evaluate the operational effectiveness of the oscillating sieving shaker, three distinct commercial powders were used: soy lecithin, potato starch, and ascorbic acid. Each sample was weighed to 10 g using an analytical balance with a precision of ±0.0001 g. The samples measured were placed on the top sieve of the assembled sieve stack.

The machine was configured to operate at 100 % power output, which corresponds to an oscillation speed of 150 revolutions per minute (RPM). The sieving process was conducted for 30 min under constant operating conditions, ensuring a consistent energy input and vibration amplitude for all samples. Although a 30-minute sieving time was used for a 10-gram sample, the relationship between processing time and sample mass is nonlinear; therefore, for other sample masses, 30 min or less could be used.

The sieving procedure was designed in accordance with the recommendations in ASTM C136/C136M-19, which define standard conditions for dry sieve analysis of powders and granular materials. These standards specify oscillation frequencies near 150 RPM, sieving durations of 15–30 min, and total sample loads not exceeding 2 kg, parameters adopted for the present configuration. Aligning the operational settings with these normative procedures ensures that the resulting particle-size distributions are comparable to those obtained from conventional laboratory analyses and validates the reproducibility of the developed oscillating sieving shaker.

Following the sieving cycle, each sieve was weighed to determine the amount of samples retained. The percentage of sample retention (%RP) on each sieve was calculated by mass difference using Eq. (1).

(1) $$\%RP=\frac{\left(SWS-SOS\right)}{\mathrm{TSW}}*100$$

Where:

%RP is the percentage of sample retained on sieve n, SWS (Sample with Sieve) is the weight of the sieve containing the retained sample (in grams), SOS (Sieve Only Sample) is the tare weight of the empty sieve (in grams), and TSW (Total Sample Weight) is the initial weight of the sample (in grams).

**Fig. 6:**
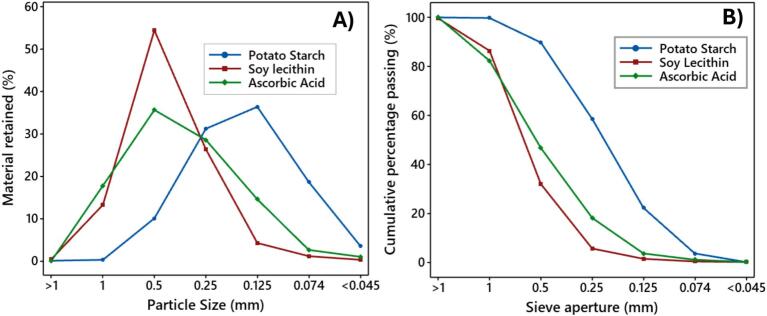
Material retained (A) and Particle size distribution curve (B) of potato starch, soy lecithin, and ascorbic acid.

This procedure was repeated five times for each material to evaluate the consistency and efficiency of the sieving process across varying particle types and physicochemical characteristics. The results obtained from these repetitions are presented in Fig. 6.

Fig. 5 presents the results of the granulometric analysis of three food-grade powders—potato starch, soy lecithin, and ascorbic acid—conducted using the low-cost oscillating sieving shaker machine.

Material retained (Fig. 5A) shows the percentage retained at each sieve aperture, illustrating distinct particle-size distributions among the three materials. Soy lecithin exhibited a peak retention of approximately 54 % at the 0.5 mm sieve, indicating a coarser granular profile. In contrast, potato starch had its highest retention (∼36 %) at 0.125 mm, suggesting a finer particle distribution. Ascorbic acid showed a more uniform spread, with a maximum of 35.6 % retained at 0.5 mm, and a gradual decrease through smaller apertures.

The particle size distribution curve (Fig. 5B) shows the corresponding cumulative particle size distribution curves, reflecting the percent finer than each sieve size. These curves confirm the effective classification provided by the shaker. Potato starch had the finest distribution, with nearly 90 % passing the 0.5 mm sieve and ∼ 22 % passing the 0.125 mm sieve. Soy lecithin exhibited a sharper drop in finer fractions, with only 1.3 % passing through 0.125 mm, while ascorbic acid displayed an intermediate profile between the two.

The particle size results obtained for each material align with reported values in the literature, confirming the analytical accuracy of the developed shaker. Potato starch powders are commonly reported with particle sizes between 62 and 278 μm for ball-milled or native granules, depending on processing method [19]. Although Soy lecithin, in its refined or vesicular forms, exhibits nanoscale dimensions (e.g., diameters exceeding 300 nm), the specific Soy lecithin employed constitutes a bulk granular material. This material characteristically appears as coarse agglomerates or waxy solids with particle side dimensions typically in the hundreds of micrometers range [20,21]., as substantiated by sieve performance evaluations. Ascorbic acid in unencapsulated crystalline form exhibits intermediate micrometric particle sizes comparable to 300–600 μm, consistent with spray-dried or powdered preparations [22]. The measured retention peaks of ∼ 0.125 mm for starch, ∼0.5 mm for lecithin, and intermediate distributions for ascorbic acid correspond closely with these literature-based expectations, validating the device’s capability to distinguish powders with distinct granulometric profiles and confirming the reproducibility of the sieving process.

Together, the retained and cumulative data demonstrate that the developed sieving system provides accurate and reproducible particle size separation across a range of food materials. This validates the equipment’s effectiveness for granulometric analysis in food science, supporting its utility as a cost-efficient alternative to commercial sieve shakers.

## CRediT authorship contribution statement

**Juan Carlos Nuñez Dorantes:** Writing – review & editing. **Mario Luna Flores:** Writing – original draft, Validation, Conceptualization. **José Roberto Grande Ramírez:** Validation, Methodology, Formal analysis. **Verónica Sanchez Flores:** Software, Project administration. **Jonathan Josue Cid Galiot:** Validation, Methodology, Formal analysis, Conceptualization. **José Ernesto Domínguez Herrera:** Writing – review & editing, Supervision, Funding acquisition, Conceptualization.

## Declaration of competing interest

The authors declare that they have no known competing financial interests or personal relationships that could have appeared to influence the work reported in this paper.
